# Mitochondrial dysfunction as a mechanism of drug-induced hepatotoxicity: current understanding and future perspectives

**DOI:** 10.18053/jctres.04.201801.005

**Published:** 2018-05-28

**Authors:** Anup Ramachandran, Ruben G.J. Visschers, Luqi Duan, Jephte Y. Akakpo, Hartmut Jaeschke

**Affiliations:** ^1^ *Department of Pharmacology, Toxicology & Therapeutics, University of Kansas Medical Center, Kansas City, KS, United States*

**Keywords:** acetaminophen, diclofenac, rifampin, isoniazid, valproic acid, herbal supplements, pyrrolizidine alkaloids, mitochondria

## Abstract

Mitochondria are critical cellular organelles for energy generation and are now also recognized as playing important roles in cellular signaling. Their central role in energy metabolism, as well as their high abundance in hepatocytes, make them important targets for drug-induced hepatotoxicity. This review summarizes the current mechanistic understanding of the role of mitochondria in drug-induced hepatotoxicity caused by acetaminophen, diclofenac, anti-tuberculosis drugs such as rifampin and isoniazid, anti-epileptic drugs such as valproic acid and constituents of herbal supplements such as pyrrolizidine alkaloids. The utilization of circulating mitochondrial-specific biomarkers in understanding mechanisms of toxicity in humans will also be examined. In summary, it is well-established that mitochondria are central to acetaminophen-induced cell death. However, the most promising areas for clinically useful therapeutic interventions after acetaminophen toxicity may involve the promotion of adaptive responses and repair processes including mitophagy and mitochondrial biogenesis, In contrast, the limited understanding of the role of mitochondria in various aspects of hepatotoxicity by most other drugs and herbs requires more detailed mechanistic investigations in both animals and humans. Development of clinically relevant animal models and more translational studies using mechanistic biomarkers are critical for progress in this area.

**Relevance for patients:**This review focuses on the role of mitochondrial dysfunction in liver injury mechanisms of clinically important drugs like acetaminophen, diclofenac, rifampicin, isoniazid, amiodarone and others. A better understanding ofthe mechanisms in animal models and their translation to patients will be critical for the identification of new therapeutic targets.

## 1. Introduction

Mitochondria are the powerhouses of the cell. They are responsible for generation of energy in the form of ATP and play important roles in cellular metabolism. While those functions were in focus traditionally, it is now recognized that mitochondria also play key roles in intracellular signaling, and probably function as an integrating platform for numerous cellular mediators, which translocate from the cytosol to the mitochondria to propagate cell signaling. Hepatocytes are one of the cell types with abundant mitochondria due to their important role in metabolism and the organelle is recognized to be a critical mediator in hepatotoxicity induced by a number of drugs including acetaminophen (APAP) and others. While the role of the mitochondria is well understood in contexts such as acetaminophen overdose and valproic acid toxicity, targeting the organelle from a therapeutic standpoint requires comprehensive information about various facets of mitochondrial function in the context of drug-induced liver injury.

## 2. Acetaminophen hepatotoxicity

APAP overdose is the leading cause of acute liver failure in the United States [1] and hepatotoxicity of the drug is due to generation of a reactive metabolite *N*-acetyl**-p**-benzoquinone imine (NAPQI). While therapeutic doses of APAP are predominantly metabolized by glucuronidation and sulfation and only a minor fraction (10%) is oxidized [2], excessive APAP levels during an overdose result in the substantially increased drug metabolism by the cytochrome P450 system to produce elevated levels of NAPQI [2,3]. While the liver has significant stores of reduced glutathione to scavenge the small amounts of NAPQI generated after a therapeutic dose ofAPAP, this is overwhelmed during an overdose, where NAPQI initially depletes liver GSH and subsequently forms adducts with cellular proteins. A major target for NAPQI protein adducts formation within the hepato-cyte is the mitochondria, which plays a central role in mediating liver injury after APAP [3]. In addition, recent evidence in mice indicates that the organelle is also important during recovery from APAP-induced liver injury [4], a clinically relevant issue, since APAP overdose patients typically present late to hospitals. These aspects will be discussed in detail in subsequent sections.

### 2.1. Mitochondrial protein adducts and APAP hepatotoxicity

Evaluation of the impact of NAPQI-protein binding on APAP hepatotoxicity in comparison to its regioisomer N-acetyl-meta-aminophenol (AMAP), which was considered to be non-toxic in mice, revealed that only an APAP overdose resulted in mitochondrial protein adducts, which were not evident after AMAP treatment [5-7]. Thus, mitochondrial protein adducts seem to be the defining feature of APAP hepatotoxicity and a dose response study of APAP toxicity *in vivo* shows that mito-chondrial protein adducts are important for the subsequent mi-tochondrial dysfunction [8]. While AMAP was considered non-toxic in mice, it has recently been shown to be hepatotoxic in primary human hepatocytes [9-11]; the toxicity still correlated with formation of mitochondrial protein adducts [10], confirming the role of mitochondrial protein adducts in APAP-induced liver injury in humans. Analysis of adducted proteins within mitochondria by proteomic techniques suggests that formation of NAPQI adducts is a targeted process, resulting in modification of specific proteins within the organelle. Since NAPQI targets cysteine residues, a global approach examining cysteine group modifications on proteins both within the matrix as well as mi-tochondrial membranes was carried out in vivo. Though specific proteins such as HMG CoA synthase were modified with inhibition of enzyme activity [12], APAP overdose did not produce global alterations in cysteine group modifications on proteins. Proteomic studies on liver cells in 3D culture also identified nu-merous mitochondrial proteins with NAPQI modifications after APAP exposure, including peroxiredoxin 6 and the voltage gated ion channel, VDAC2 [13]. However, in spite of extensive investigations over the years, none of these protein modifications have been shown to be singularly responsible for induction of subsequent mitochondrial dysfunction and hepatocyte necrosis. For example, modifications on glutathione peroxidase and an ATP synthase subunit were identified by proteomics in vivo [14], and glutathione peroxidase activity was also inhibited after APAP treatment [15]. This would suggest that dysfunction of glutathione peroxidase by NAPQI adduct formation could be responsible for the downstream events. Interestingly, mice with a genetic deficiency of glutathione peroxidase did not demonstrate any exacerbation of liver injury after APAP [16], indicating that these modifications are most likely a consequence, rather than a cause of mitochondrial dysfunction. However, while individual protein modifications are unlikely to influence downstream events, mitochondrial adduct formation as a whole is important for subsequent steps in toxicity, since NAPQI binding to mitochondrial proteins correlates with toxicity [17]. Taken together, the current data indicate that formation of mitochon-drial protein adducts on a number of different proteins, probably beyond a certain threshold, is critical for initiating mitochondrial dysfunction and subsequent cell signaling ultimately resulting in hepatocyte necrosis.

### 2.2. Mitochondrial oxidative and nitrosative stress in APAP-induced liver injury

In addition to producing ATP, mitochondrial respiration is also an important source of reactive oxygen species (ROS), which are usually scavenged by anti-oxidant enzymes such as manganese superoxide dismutase (SOD2) to prevent cellular damage. Modulation of mitochondrial bioenergetics can have wide ranging effects on a pathophysiology, and is one of the mechanisms of protection by the APAP antidote *N*-acetylcysteine (NAC), which, in addition to replenishing glutathione stores, can also support mitochondrial energy metabolism [18]. Formation of NAPQI protein adducts on mitochondrial proteins increases free radical production [19] and decreases mitochondrial respiration in vivo [20]. Recent evidence from both in vitro as well as in vivo experiments also indicates that APAP interferes with the formation of mitochondrial respiratory super complexes via the mitochondrial negative regulator MCJ, which could be the cause ofdecreased production ofATP and increased generation ofROS [21]. The role of free radicals such as superoxide in APAP-induced hepatotoxicity is also illustrated by the exacerbation of injury in mice with partial SOD2 deficiency [22,23] as well as the protection against APAP hepatotoxicity afforded by the mitochondria-targeted SOD-mimetic mito-tempo *in vivo* [24]. Another factor which could influence superoxide production within mitochondria is oxygen tension. Primary mouse hepato-cytes cultured at 10% oxygen demonstrated reduced mitochon-drial oxidant stress, peroxynitrite formation and cell death compared to those cultured at room air (21% oxygen) [19]. Elevated superoxide within mitochondria can either dismutate to hydrogen peroxide or react with nitric oxide within the mitochondria to produce the highly reactive peroxynitrite and in quantitative terms, the fate of superoxide radicals will depend on the competition between these two reactions [25]. Generation of peroxyni-trite results in nitration of tyrosine residues on proteins [26] and examination of nitrotyrosine levels as a marker of peroxynitrite formation indicated significant elevations exclusively within liver mitochondria within 1 hour after a dose of 300 mg/kg APAP *in vivo* [27]. This indicates that superoxide was reacting with nitric oxide within the mitochondria early after generation of protein adducts. While nitrotyrosine adducts are also evident later in hepatocytes and sinusoidal endothelial cells [28,29], the generation within mitochondria seems to be the initial trigger causing further downstream events [27]. But what is the source of nitric oxide for this peroxynitrite production? Nitric oxide can be synthesized by 3 main isoforms of nitric oxide synthase (NOS), and while all of them have been implicated at various times in the APAP-induced NO generation [30-32], peroxynitrite production seems to be independent ofinducible NOS (iNOS) in vivo [29,33]. A number of recent investigations suggest a predominant role for neuronal NOS (nNOS) in APAP-induced hepatic injury, with mice lacking nNOS being protected from APAP induced hepatotoxicity [32]. Treatment of primary mouse hepatocytes with an inhibitor of nNOS, namely (N-[(4S)-4-amino-5-[(2-aminoethyl)amino]pentyl]-N'-nitroguanidinetris (trfluoroa-cetate)) (NANT), was also protective [34], as was use of triflu-operazine (TFP), a calmodulin antagonist that inhibits calcium induced nNOS activation both in vitro as well as in vivo [35]. This is relevant, since APAP has been shown to increase cy-tosolic calcium levels in isolated hepatocytes by inhibition of plasma membrane calcium-ATPase [36], as well as preventing mitochondrial calcium uptake *in vivo* [5]. Also, nNOS has been identified in hepatocytes [37] and NO preferentially partitions into lipid membranes [25] enabling its reaction with superoxide generated within mitochondria. Protein nitration by peroxynitrite can significantly influence protein function [25] and a major consequence of mitochondrial peroxynitrite formation after APAP overdose *in vivo* is nitration of mitochondrial anti-oxidant proteins such as manganese superoxide dismutase [38] as well as mitochondrial DNA damage [27]. The critical role of peroxy-nitrite in mediating downstream events in APAP hepatotoxicity is evident by the benefit of its scavenging by molecules such as glutathione, which replenished mitochondrial levels ofGSH and scavenged peroxynitrite [16]. Glutathione supplementation thus provides both early benefit [16] as well as protection after delayed treatment [39,40], with enhanced liver regeneration [40] in vivo. Overall, it is now clear that mitochondrial generation of peroxynitrite is a central event which has significant influence on various cell signaling pathways, ultimately resulting in hepa-tocyte necrosis.

### 2.3. Mitochondria as a signal integration platform after APAP

An early consequence of APAP-induced mitochondrial oxi-dative/nitrosative stress is activation of c-jun *N*-terminal kinase (JNK) in the cytosol, the prevention of which by anti-oxidants in vivo confirms its dependence on mitochondrial free radical ge-neration [33,41]. JNK activation is an early event after APAP overdose, occurring in the cytosol within 1 hour of treatment of mice with 300mg/kg APAP [42] and seems to be influenced by the apoptosis signal-regulating kinase 1 (ASK1) since mice lacking ASK1 were protected against APAP-induced JNK acti-vation [43]. A small molecule ASK1 inhibitor also prevented JNK activation and protected against liver injury when admi-nistered prior to APAP or shortly after APAP [42]. Since acti-vation of ASK1 requires oxidation and detachment of its bin-ding partner thioredoxin 1 [44], this could be the mechanistic interface between mitochondrial oxidant stress and ASK 1 activation, since thioredoxin oxidation occurs within mitochondria after APAP overdose in vivo [45] and activities of thioredoxin 1 & 2 have been shown to be modified by NAPQI [44]. In addition, the mixed-lineage kinase 3 (MLK3) is also activated by oxidant stress during APAP hepatotoxicity in vivo [46] which, along with ASK1, phosphorylates JNK through the MAPK2 kinase MKK4 [33,41,47]. Phosphorylation and activation of JNK within the cytosol subsequently results in its translocation to the mitochondria [41] by binding to a docking protein Sab on the outer mitochondrial membrane [48]. The binding to and phos-phorylation of Sab by p-JNK on the outer mitochondrial membrane leads to release of protein tyrosine phosphatase nonreceptor type 6 (SHP1) from Sab in the inside of the mitochondrial outer membrane and subsequent decrease in mitochondrial respiration [49] and amplification of oxygen free radical and peroxynitrite formation [33]. The fact that this is a stepwise process: JNK activation → Translocation to mitochondria → Amplification of mitochondrial oxidant stress, is illustrated by the fact that inter-vention with the anti-diabetic drug metformin in mice does not influence JNK activation or translocation, but attenuates ampli-fication of mitochondrial oxidant stress, probably by inhibition of respiratory complex I [50]. While typically JNK activation and translocation to mitochondria along with amplification of free radical generation results in irreversible mitochondrial dys-function, it is now evident that this is dependent on the initial dose of APAP. Exposure to lower APAP doses of 150 mg/kg in mice resulted in transient JNK activation, with reversible mito-chondrial dysfunction [8]. In addition to JNK, other cytosolic proteins such as Bax also undergo translocation to mitochon-dria early after APAP overdose in mice [51,52], though this does not seem to influence mitochondrial oxidant stress and peroxy-nitrite formation [51], but may be directly involved in regulation of subsequent alterations such as the mitochondrial permeability transition, detailed below. In addition to JNK and Bax, trans-location of cytosolic glycogen synthase kinase-3// (GSK-3//), a major regulator of glycogen synthase, to mitochondria has also been shown early after APAP overdose, and silencing GSK-3/ protected against APAP hepatotoxicity in vivo [53]. Inhibition of GSK 3 also accelerated liver regeneration after APAP overdose in mice [54]. In addition to proteins, translocation of metals such as iron to the mitochondria from lysosomes also occurs in hepatocytes *in vitro* after an APAP overdose [55] and the influ-ence of this will also be detailed in the next section.

### 2.4. Acetaminophen induced mitochondrial permeability transition and nuclear DNA fragmentation

The amplification of oxidative and nitrosative stress due to phospho-JNK-mediated inhibition of mitochondrial respiration results in a catastrophic collapse of mitochondrial function, with activation of the mitochondrial permeability transition (MPT) pore. This is an abrupt increase in permeability of the mitochon-drial inner membrane in response to excessive oxidative stress, which causes the leakage of mitochondrial contents up to a size of 1.5 kDa into the cytosol [56]. Induction of the MPT results in a loss of the mitochondrial membrane potential due to dissipation of the proton gradient and accompanying collapse of mi-tochondrial ATP production, ultimately resulting in cell necrosis [57-60]. It is generally agreed on that the MPT is caused by the opening of a large channel (mitochondrial permeability transition pore), which is composed of various components, including Bax and Bak [61,62] on the mitochondrial outer membrane and subunits of the ATP synthase on the inner membrane [56]. Ad-ditional regulatory components include cyclophilin D [63], mo-dulation of which can influence APAP hepatotoxicity in a dose dependent manner [57,59,60]. While inhibition of cyclophilin D provided partial protection *in vitro* [57] and prevented APAP-induced liver injury at a moderate overdose of 200 mg/kg *in vivo* [59], no protection was seen at a higher APAP dose of 600 mg/kg [60]. This dose-dependent modulation of the MPT was corrobo-rated *in vivo* by another study using intravital microscopy, where a dose of 150mg/kg APAP resulted in transient activation of JNK and reversible loss of membrane potential without cell death [8], suggesting that activation of the MPT could be restricted to certain mitochondria within cells without propagation to cell death. Mitochondria are also a central organelle for iron metabolism [64] and iron can also induce activation of the MPT [65]. Translocation of iron from lysosomes to mitochondria does occur after APAP overdose [55], which is probably facilitated by lysosomal instability seen under these conditions [66]. Interestingly, while physiological iron import into mitochondria is thought to be mediated by the mitoferrin proteins on the inner membrane [64], transport after APAP overdose seems to be occurring through the calcium uniporter, since its inhibition prevented mitochon-drial oxidant stress and loss of membrane potential [67]. As a consequence of the MPT, a large variety of mitochondrial proteins are released into the cytosol, including endonuclease G and apoptosis-inducing factor (AIF), both of which contain a nuclear localization sequence [68]. These proteins thus translocate to the nucleus after APAP overdose [69] causing DNA fragmentation, and subsequent cellular necrosis. Their importance, especially of AIF, in mediating these final steps is confirmed by the reduced DNA fragmentation and protection against liver injury seen in mice with partial AIF deficiency after APAP [70].

### 2.5. Role of mitochondria in recovery after APAP-induced liver injury

Though mitochondrial protein adduct formation, oxidati-ve/nitrosative stress and ultimately the membrane permeability transition play critical roles in cellular necrosis as detailed above, recent evidence implicates the organelle in recovery subsequent to liver injury [4]. APAP-induced cell death is not uniform throughout the liver, with necrosis predominantly seen in cells around the central vein, with progressively lesser injury towards the periportal area [71]. Activation of mitochondrial biogenesis in cells surrounding areas of necrosis was recently found to be important for recovery after APAP-induced liver injury, with pharmacological enhancement of biogenesis facilitating regeneration and recovery [4]. Mitophagy, the process by which damaged mitochondria are removed selectively by autophagy is another feature of recovery after APAP induced liver injury [71,72], with inhibition of mitophagy sensitizing mice to APAP hepato-toxicity [73,74].

Thus, mitochondria play a critical role in hepatocyte necro-sis induced by an APAP overdose, starting from formation of protein adducts on the organelle, generation of oxidative and ni-trosative stress, which ultimately activates the induction of the MPT and subsequent nuclear DNA fragmentation. In addition to its role in cell signaling during the early injury phase, mito-chondrial biogenesis in cells surrounding the necrotic area is an important determinant of liver regeneration and recovery after an APAP overdose.

## 3. Diclofenac hepatotoxicity

Diclofenac, one of the most widely used non-steroidal anti-inflammatory drugs (NSAIDs), is commonly used to treat pain and swelling associated with rheumatic disorders [75]. Its efficacy for treating pain and inflammation is due to inhibition of cyclooxygenase (prostaglandin endoperoxide synthase), the enzyme mediating production of prostaglandins and thromboxane A2 [76]. In general, diclofenac is well tolerated, with the main concerns regarding the use of diclofenac being the possibility of bleeding from both the operative site (because of the inhibition of platelet aggregation) [77], and from the upper gastrointestinal tract (especially in patients stressed by surgery, the elderly, and frail or dehydrated persons). Other serious adverse events include acute renal injury, heart failure, adverse reproductive outcomes [78] and liver injury [79]. Diclofenac is the most frequently reported NSAID showing side effects predominantly related to hepatocellular (centrilobular), mixed and cholestatic type of liver injury, which leads to liver failure [80]. Diclofenac liver toxicity in humans is idiosyncratic [81], and although various mechanisms for diclofenac-induced liver injury have been proposed, none have been fully understood. During diclofenac-induced liver damage, a rapid and concentration-dependent cellular ATP depletion (preceding overt cell injury) was observed, suggesting an important role for mitochondria in this process, and several studies have shown that both diclofenac metabolites and their parent compounds can cause hepatocyte mitochondrial dysfunction leading to hepatocyte damage.

### 3.1. Diclofenac metabolites and their influence on mitochondria

In hepatocytes, diclofenac is extensively metabolized by both phase I and phase II reactions, and several reactive metabolites have been shown to inhibit ATP synthesis. In human liver microsomes, the major oxidative metabolic pathway (the phase I reaction) of diclofenac is the formation of 4'-hydroxydiclofenac (4'-OHdic) by CYP2C9 [82,83]. Formation of 5-hydroxydiclofenac (5-OHdic), 3'-hydroxydiclofenac, 4'**,** 5-dihydroxydiclofenac, and 3'-hydroxy-4'-methoxydiclofenac has also been reported in humans [84,85]. In the rat, 4 -OHdic together with 5-OHdic are the major urine metabolites [86]. The metabolite 5-OHdic and its oxidized form *N*,5-dihydroxydiclofenac **(N**,5-(OH)_2_ dic) can oxidize mitochondrial NADPH and decrease ATP [87]. Both 4 -OHdic and 5-OHdic show potent ATP synthesis inhibition, with 4 -OHdic being the more potent inhibitor [88]. Studies by Ponsoda et al suggested that diclofenac toxicity required oxidative metabolism of the drug, and hepatocyte injury was preceded by a decrease in ATP levels [89], suggesting mitochondrial ATP production as a target in hepatocyte toxicity. The fact that diclofenac metabo-lism, especially through cytochrome P450 was required for cy-totoxicity was further re-iterated by studies by Jurima-Romet M et.al in rat primary hepatocytes [90]. Hepatotoxicity of oxida-tive metabolites ofdiclofenac was confirmed by use ofP450 inhibitors, which decreased hepatocyte damage and mitochondrial impairment [87,91]. In the phase-II conjugation pathway, the bio-activation of diclofenac to diclofenac 1-O-acyl glucuronide (DicGluA) is catalyzed by UGT in hepatocytes, though DicGluA can also transacylate glutathione to form diclofenac glutathione thioester (DicSG) [92]. Both DicGluA and DicSG show inhibi-tion of ATP synthesis, with DicGluA exhibiting stronger inhibi-tion than DicSG [88]. These studies highlight the requirement for metabolism of diclofenac in toxicity and suggest mitochondria to be critical cellular targets for this toxicity by compromised generation of ATP.

Several pathways could account for the inhibition of ATP synthesis, including opening of the MPT pore, which may lead to the loss of the mitochondria membrane potential (ψψm) re-quired for synthesis of ATP [93]. The mechanism of opening of the MPT pore by diclofenac metabolites is not fully under-stood, though ROS and increase in cytosolic calcium have been suggested to be involved [88,94,95]. Oxidative intermediates of diclofenac were associated with increases in cytosolic calcium and the highly selective calcium chelator BAPTA greatly attenuated diclofenac-induced cell injury [88]. The direct role of diclofenac metabolites in mediating induction of MPT through increased cytosolic calcium is also suggested by the lack of effect of the MPT pore inhibitor cyclosporin A on calcium levels and the prevention of cytosolic elevation of calcium by blockade of CYP2C9-mediated oxidative bioactivation of diclofenac [88].While the exact mechanisms by which diclofenac metabolites increase calcium are not well characterized, one possibility could be ROS produced by the reactive metabolites of diclofenac. Since the 4 -hydroxylated and 5-hydroxylated diclofenac metabolites canbe further metabolized to the respective intermediates [96], ROS may be generated through redox cycling via the semi-quinone radical. Similarly, a minor metabolite *(N*,5-dihydroxy diclofenac) may induce oxidant stress through consumption of NADPH [87]. Thus, based on available literature, both phase I and phase II metabolites are implicated in redox-cycling to produce oxidative stress, inducing opening of the MPT pore and dissipation of transmembrane potential resulting in depletion of ATP. On the other hand, it is possible that diclofenac metabolites may directly affect mitochondrial protein function by forming adducts to cause uncoupling of respiration, MPT pore opening and subsequent ATP depletion. In addition, oxidative metabolites may be involved in elevation of cytosolic calcium levels, which could also play a role in induction of the MPT and triggering of cell death [95]. Furthermore, opening of the MPT leads to the release of pro-apoptotic factors such as cytochrome c, which contributes to the activation of caspase-9 and -3, and leads to apoptotic cell death [97].

### 3.2. Influence of diclofenac on functional parameters of mitochondria

Apart from diclofenac metabolites, the parent compound has also been demonstrated to target mitochondria and induce mito-chondrial dysfunction [98-100]. Specifically, in isolated liver mitochondria, diclofenac readily inhibits ATP synthesis and induces the MPT [94,97], leading to a collapse of the mitochon-drial transmembrane potential (A*_m_ ). In this context, diclofe-nac, which is a lipophilic and weak acidic compound, works as an uncoupler of oxidative phosphorylation [101], translocating protons across the inner mitochondrial membrane to cause uncoupling. In addition, some studies suggest that the uncoupling effect of diclofenac stimulates mitochondrial respiration and enhances superoxide generation, with the increased ROS oxidizing membrane thiols, which play important roles in opening of the MPT [94]. Diclofenac was also shown to decrease the oxygen consumption rate in human hepatocytes [102] as well as in isolated rat liver mitochondria, accompanied by increased mitochon-drial swelling [103]. In addition, diclofenac caused a decrease in ATP levels in human hepatocytes [104]. While detailed mechanistic studies on molecular targets of diclofenac within the respiratory chain are scarce, studies in saccharomyces cerevi-siae indicate that diclofenac inhibits respiration by interfering with Rip1p and Cox9p in the respiratory chain, resulting in ROS production that causes cell death [105]. Analysis of molecular mediators involved in mitochondrial MPT pore opening indicated that diclofenac induced early activation of Bax and Bak in immortalized human hepatocytes (HC-04), and caused mito-chondrial translocation of Bax [106]. This was accompanied by activation of Bid, which was sensitive to calcium chelation, suggesting that the calcium-Bid-Bax-MPT axis is a critical pathway in diclofenac induced cell death [106].

Thus, diclofenac induced MPT pore opening and subsequent decrease of mitochondria membrane potential (ψψm), ATP depletion and apoptotic cell death was reported in metabolizing hepatocytes [93,107,108]. The events following MPT pore opening after diclofenac causing hepatocytes apoptosis have been well been studied in rat and human hepatocytes. Apoptosis was observed after exposure to sub-cytotoxic concentrations of diclo-fenac, mediated by opening the MPT pore and increased ROS, with activation ofcaspase 3, 8 & 9. These changes were prevented by anti-oxidant treatment, implicating mitochondrial ROS in caspase activation and downstream induction of apoptosis [97,99]. It was also recently shown that diclofenac treatment decreased mitochondrial membrane potential in both rat and human hepatocytes in vitro, accompanied by mitochondrial fragmentation due to decrease in the mitochondrial fusion protein Mfn1 [104]. Rats treated chronically with diclofenac (5 or 10 mg/kg) for 15 days also showed enlarged mitochondria with irregular and ruptured mitochondrial membranes [109].

In summary, diclofenac and its metabolites induce hepatic mitochondrial dysfunction through a number of different mechanisms (1) direct effects on the mitochondrial inner membrane; (2) uncoupling of respiration by proton shuttling or direct effect on respiratory chain complexes (3) changes in cytosolic calcium, which, along with compromised mitochondrial function facilitate the MPT pore opening (4) decrease of mitochondrial transmembrane potential, followed by ATP depletion; and (5) alterations of mitochondrial morphology and dynamics.

## 4. Rifampin and isoniazid hepatotoxicity

Tuberculosis (TB) is a major health problem worldwide but is a curable disease by treatment with anti-tuberculosis drugs [110]. Rifampin (RMP) and Isoniazid (INH) are the two most potent first line antibiotics that are used in combination to treat tuberculosis [110,111]. While an acute overdose of anti-TB drugs leads to neurotoxicity, chronic therapeutic treatment is the most common cause of liver injury [112,113]. While hepatotoxicity due to RMP and INH is well documented [110,114,115], there are not many studies detailing the mechanisms behind liver injury induced by these drugs. Many risk factors such as age, alcohol consumption, gender and underlying diseases such as hepatitis B and C or cirrhosis could make TB patients undergoing anti-TB treatment more susceptible to RMP and INH hepatotoxicity [116-118].

RMP is bio-activated and detoxified in the liver [110,111]. Rifampin particularly induces many drug metabolizing enzymes such as CYP1A2, 2C9, 2C19 and 3A4 and therefore increases chances of liver injury caused by additional anti-TB drugs [110] as well as other drugs like APAP [119]. Biotransformation of RMP leads to formation of a reactive toxic intermediate, dea-cetyl rifampin, which can bind to cellular macromolecules and cause liver injury [116]. Rifampin hepatotoxicity is initially characterized by an increase in plasma AST and ALT activities as well as bilirubin levels within 1 to 6 weeks of therapy initiation [120]. During the course of hepatotoxicity, AST and ALT levels return to baseline. Thus, ALT and AST levels are good markers for early detection of potential adverse liver effects to RFP [121]. However, only bilirubin can serve as a long term surrogate for liver injury because it remains elevated and can be detected at later time points of RMP-mediated liver injury [122].

INH is mainly metabolized by CYP2E1 and detoxified in the liver [110]. Similar to RMP, liver injury is mediated by a toxic reactive intermediate, acetylhydrazine. Resistance to INH occurs frequently and requires its combination with RMP (RMP-INH) [123]. Single treatment of IND causes liver injury, which occurs much later than single treatment of RMP. INH-mediated liver injury manifests 2 weeks to 6 months after the initial treatment [110] compared to 1-6 weeks with RMP as mentioned above. Similar clinical outcomes (hypersensitive allergic reaction or necrotic cell death) occur with single treatment ofINH [110] when compared to RMP. Severe (liver transplant) and fatal (death) outcome is associated with increased jaundice in RMP or INH mediated liver injury [124]. Their reactive metabolites may also form a neo-antigen which induces a hypersensitive immune-allergic reaction characterized by clinical symptoms such as skin rash, nausea, anorexia, abdominal discomfort and fatigue [125].

In case of co-treatment with RMP and INH, the issue is that RMP is a potent inducer of CYP2E1, which is the major enzyme responsible for metabolism of INH [126,127]. Thus, at chronic therapeutic doses, the combination therapy results in increased production of the toxic acetylhydrazine and hydrazine metabolites in the liver. This increase in toxic RMP-INH metabolites has been shown to affect cellular homeostasis by decreasing hepatic GSH in mice [128], and reducing mitochondrial ATP production [128]. Significant liver damage due to synergistic action of RMP-INH can be prevented by early detection of hepatic injury and NAC treatment [129].

### 4.1. Role of mitochondria in RMP/INH mediated hepatotoxicity

Analysis of metabolites formed after high and long term dosage of INH in rats revealed that INH hepatotoxicity is due to INH induced mitochondrial dysfunction/damage [130]. In HepG2 cells, INH metabolites also cause manganese superoxide dismutase [127,131], catalase, and glucose-6-phosphate dehydrogenase modifications [132]. These altered antioxidant functions lead to the loss of hepatocyte ability to efficiently detoxify acetylhydrazine and hydrazine. As a result, acetylhydrazine and hydrazine bind covalently with hepatic mitochondrial proteins and inhibit their function [119,128,133] while decreasing ATP production and nicotinamide adenine dinucleotide levels [134]. INH induced hepatotoxicity studies in both rats and humans reveal that there is a robust connection between isoniazid INH metabolic activation by CYP2E1, and ROS production and mito-chondrial dysfunction [119,133]. This is also consistent with data from RMP-INH co-treatment, showing that the drugs increase hepatic oxidant stress and trigger onset of the MPT [128]. The INH reactive metabolite, hydrazine, was shown to directly inhibit the activity of solubilized complex I in mouse hepatocy-tes while causing mitochondrial oxidant stress [135]. This INH induced mitochondrial oxidative stress affects mitochondrial dynamics in various ways [136]: INH induced mitochondrial ROS and reduced the membrane potential in rat liver mitochondria [115]. This was similarly demonstrated in HepG2 cells in which INH not only reduced the membrane potential but also indu-ced mitochondrial swelling [136] and led to changes in mito-chondrial structure and its perinuclear localization [136,137]. In INH treated lymphoma cells, the fluorescent probe JC-1 revealed a breakdown of the mitochondrial membrane potential [138]. During INH hepatotoxicity, hepatocytes try to survive by maintaining mitochondria homeostasis via mitochondrial biogenesis. However, INH caused impairment of mitochondrial biogenesis in HepG2 cells [136,139]. Amongst the multitude of factors controlling mitochondrial biogenesis, peroxisome proliferator-activated receptor gamma coactivator 1-alpha, PGC1**a,** plays a central role in signal transduction. However, PGC1**a** was inhibited by INH in HepG2 cells [136,139]. PGC1**a** activates many downstream transcription factors. INH inhibits PGC1**a** and sir-tuin 1 (SIRT1), as well as the nuclear respiratory factor 1 (NRF1)[136,139]. Zhang et. al found that INH decreased expression of mitochondrial protein COX IV , resulting in lower mitochon-drial mass [136]. INH also decreased levels of the fusion pro-tein MFN2 as well as the fission protein DRP1 [136]. This sug-gests that both aspects of mitochondrial dynamics, namely fusion and fission are impaired by INH. Decrease of DRP1 expression by INH was shown to be tightly linked with mitochon-dria mediated onset of apoptosis [136]. Further evidence shows that the mitochondrial oxidative stress induces apoptotic cell de-ath after RMP-INH treatment in mice [128], HepG2 cell and AHH1 cell lines [132,138]. The concept that RMP-INH mediated apoptotic cell death of hepatocytes is mediated via mi-tochondrial cytochrome c release and mitochondrial mediated caspase activation is supported by a number of studies, which show that RMP-INH mediated mitochondrial ROS production inhibits Nrf2 function [140] and induces pro-caspase-8 & 10 activation in rats [126,140]. Furthermore, altered mitochondria-mediated changes in Bcl-2/Bax content, mitochondrial release of cytochrome c and caspase activation were also all shown to be responsible for INH- induced mitochondria mediated apopto-sis in HepG2 cells [132]. Detection of cytochrome c protein in the cytosol of mouse hepatocytes treated with RMP-INH also re-vealed its leakage from mitochondria [128]. All of these studies support a RMP-INH mediated mitochondrial mode of apoptotic cell death.

### 4.2. Role of nuclear hormone receptors

RMP is a well-known ligand for nuclear receptors such as the pregnane X receptor (PXR), and high dose RMP has been shown to stimulate nuclear translocation of mPXR in the liver of mice by indirect activation, resulting in the transactivation of Cyp3a11 and other PXR-target genes [141]. In addition, hepatic PXR was rapidly activated in RMP treated mice, with transcripti-onal upregulation of the PXR target, PPAR**7,** ultimately resulting in increased uptake of fatty acids from circulation into the liver [142]. However, while RMP activation of PXR and its effect on cytochrome P450, glucuronosyltransferases and p-glycoprotein activities to modulate metabolism of other drugs is well known [143], mitochondrial targets subsequent to PXR activation in the context of RMP hepatotoxicity have not been extensively studied. Enzymatic analysis of CYP3As and CYP2B in mitochondria from pig liver however, showed upregulation in response to RMP [144].

### 4.3. Synergistic effects of rifampin and isoniazid

Since RMP and INH are each individually metabolized by different drug metabolizing enzymes, their combination creates two different sets of toxic reactive metabolites. This is most likely overwhelming for hepatocytes and may hasten the onset of hepatotoxicity and irreversible acute liver failure (ALF) [120]. However, the exact mechanism of RMP/INH hepatotoxicity has not yet been completely elucidated. Genetic and other risk fac-tors affect drug metabolism or clearance enzymes and determine the intracellular concentration of hepatic reactive intermediate [116]. Within the literature, there is a consensus that RMP/INH phase I hepatic metabolism and failure of phase II hepatic eli-mination leads to toxic accumulation of reactive intermediates [116]. Unable to be eliminated, the elevated levels of RMP/INH reactive intermediates can bind cellular and mitochondrial pro-teins, cause mitochondrial dysfunction and induce cellular stress due to ROS production. This cellular stress causes liver injury by triggering either an immune response or hepatic apoptosis/-necrosis. In most cases, withdrawal of the RMP/INH resolves the injury [110]. However, there are reported cases that resulted in death of TB patients or the need for liver transplantation to survive [145-147]. Based on investigation of the RMP-INH mediated hepatotoxicity, mitochondrial oxidative stress seems to play a life-threatening role. RMP was found to accumulate in the liver where it induces cell death and fibrosis [148]. Changes in mitochondrial morphology, excessive ROS production and cyto-chrome c release were also reported [148]. An evaluation of the mechanisms of INH-induced mitochondrial toxicity using rat liver mitochondria showed significant glutathione oxidation, ATP depletion and lipid peroxidation [115]. A mouse study found that impairment of mitochondrial complex I amplifies mitochondrial dysfunction and precipitates INH-induced hepatocellular injury [135]. Although, RMP/INH seems to affect components of the mitochondrial electron transport chain, lipid peroxidation and mitochondrial membrane potential, there is still a need for ad-ditional investigations to elucidate the exact mechanism of mi-tochondrial dysfunction in RMP-INH-mediated liver injury.

## 5. Anti-epileptic drug hepatotoxicity

According to the Center for Disease Control and Prevention, 1.2% of the population of the United States has epilepsy, which primarily affects children [149]. Treatments with anti-epileptic drugs (AED) are the major clinical approach to management of epilepsy [150,151]. AEDs include valproic acid, hydantoin derivatives, barbiturates, benzodiazepines, succinimides, gamma amino butyric acid (GABA) precursors and analogues, as well as inhibitors of N-methyl-D-aspartate (DMDA) receptors [151]. Since AED administration is not a cure for epilepsy and is only prescribed as preventive medication to manage seizure occurrence [152], careful selection and administration of AEDs is crucial for their effectiveness and management of epilepsy [151]. Also, AED therapy is often lifelong due to the aim of improving the quality of life of patients [152], and this translates to a large number of prescriptions for the drug, increasing the possibility of development of adverse effects in the CNS and liver [153]. Though rare, even at therapeutic doses, chro-nic AED treatment has a significant potential to cause hepato-toxic effects especially in children and the consequence can be either death or need for liver transplantation due to AED-induced acute liver failure (ALF) [154]. In fact, AEDs are the third most common cause of liver transplantation due to drug-induced ALF [112]. In spite of this, due to the importance of AED at managing epilepsy and seizure occurrence, the potential hepatotoxicity of commonly used AED are accepted clinically with the hope that early detection of adverse liver effects will allow discontinuation of that particular drug and treatment with an alternate drug once the hepatotoxicity has resolved [154]. Thus, it is important to understand the mechanism of hepatotoxicity connected with AEDs in order to make the appropriate choice of suitable AEDs for epilepsy treatment.

### 5.1. AED metabolism

AEDs are metabolized in the liver and hepatic biotransfor-mation is the main route of elimination of the drug from the body [155]. As a general mechanism of toxicity, AEDs are bio-transformed into highly reactive metabolites, which if not detoxified by GSH, bind and damage critical hepatic macromo-lecules, such as mitochondrial proteins [155]. Carbamazepine and phenytoin exert their toxicity via hepatic metabolism into a highly toxic arene oxide intermediate [154]. Though the exact mechanisms behind AED hepatotoxicity are not well understood, binding of the reactive metabolite to GSH and mitochondrial proteins, compromises hepatocytes' antioxidant capacity, and induces a mitochondrial oxidant stress [156]. Thus, recent studies have postulated that mitochondrial dysfunction plays a major role in AED-induced liver injury [157-160]. AED-mediated hepatotoxicity can lead to two different outcomes: the first one is hepatocyte necrosis and acute liver failure, while the second one is formation of a neoantigen, which induces a hypersensitive immunoallergic reaction triggering severe hypersensitive symp-toms such as fever and skin rash [154].

### 5.2. Prognosis of AED-mediated hepatotoxicity

Among the common AEDs, carbamazepine, phenytoin, val-proic acid and barbiturate are predominantly reported to have the worst adverse effects on the liver [113,161]. A series of published case reports comparing patients with AED-induced liver injury who died or had a liver transplant with those who recovered from liver injury [153] showed that carbamazepine and valproic acid had the worst prognosis after ALF in children under the age of 10 [162]. However, since phenytoin and carbamazepine-induced hepatotoxicity takes an average of 4 weeks to develop after the initial treatment [154], these adverse effects can be re-versed by simply recognizing liver injury early, discontinuing treatment of AED and treating hepatotoxicity with the hepatic antioxidant used for APAP-mediated hepatotoxicity, NAC [154]. In that case, epileptic patients recover and survive. Ho-wever, failure to withdraw the AED prolongs liver injury and can lead to ALF with severe and irreversible clinical outcomes. Therefore, early detection of liver injury seems to determine whether patients recover/survive or require a liver transplant/-die. This is supported by the analysis of blood and liver biop-sies from patients with phenytoin and carbamazepine-mediated liver injury [153]. Liver injury markers such as hepatic necrosis, AST and ALT activities in the blood were significantly more elevated in patients who died or underwent liver transplantation than in patient who survived. On the contrary, immunoreacti-vity surrogates such as inflammation, and peripheral and liver eosinophilia, were significantly elevated in patients who survived [153]. This suggests that cases associated with irreversible ALF and death show metabolically-induced liver injury and mi-tochondrial dysfunction while cases associated with recovery indicate metabolically-induced hypersensitivity reactions. Since mitochondrial oxidant stress is well known to trigger hepato-cyte necrosis [3,33,163,164], these organelles may also be critical cellular targets for AED-mediated hepatotoxicity.

### 5.3. Mitochondrial dysfunction in AED-induced hepatotoxicity

Maintenance of mitochondrial function involves interaction of multiple complex pathways [156,165], which can be inhibited by AEDs to compromise normal mitochondrial function [156]. The mechanisms behind AED-mediated mitochondrial dysfunction in the case of drugs such as carbamazepine, phenytoin and valproic acidare generally well studied [156] and will be detailed below. Studies *in vitro* and in animals [156] and pilot studies in patients [149] have reported many different mecha-nisms for AED-mediated mitochondrial dysfunction.

### 5.4. Valproic acid

Valproic acid (VPA) was found to induce a statistically significant decrease of respiratory complex I activity in crude mito-chondrial fractions isolated from the pig brain [166]. Investigation of the effect of VPA on mitochondrial activity in the human hepatoma cell line, HepG2, showed that VPA treatment reduced oxygen consumption and ATP levels [167]. VPA treatment also reduced expression of DNA polymerase gamma, which plays a major role in mitochondrial DNA (mtDNA) replication and re-pair [168], as well as mtDNA levels in HepG2 cells [168]. VPA has been shown to significantly diminish mitochondrial mem-brane potential in HepG2 cells [167]. Treatment with VPA induced hepatic microvesicular steatosis accompanied by decreased respiratory control ratio, increased mitochondrial swelling and altered calcium flux in hepatic mitochondria [169]. VPA also increased the level of ROS and inhibited the antioxidant function of superoxide dismutase (SOD) in a murine hepatic microsomal system and in HepG2 cells [167,168,170]. In hepatocyte like cells differentiated from fibroblasts obtained from patients with an intractable epilepsy condition, VPA induced a mitochondrial dependent apoptosis [168]. This finding was also supported by an *in vitro* study in HepG2 cells [167], where VPA led to morphological abnormalities in mitochondria, and in addition, the levels of optic atrophy 1, which keep cristae junctions tight, were reduced.

Patients with Alpers-Huttenlocher syndrome (AHS), which is a neuronal mitochondrial disease, have increased susceptibi-lity to VPA induced hepatotoxicity. An interesting report in he-patocyte like cells differentiated from fibroblasts obtained from patients with AHS, demonstrated that VPA-induced mitochon-drial dysfunction leads to spontaneous bursts of ROS generation. This is likely due to VPA mediated opening of the MPT pore. This intermittent ROS discharge leads to mitochondrial cy-tochrome c release and caspase 9 activation, which triggers mitochondria mediated apoptosis [168,171]. Thus, VPA-induced hepatotoxicity is characterized by excessive mitochondrial dependent apoptosis [168]. During VPA-induced toxicity, the mi-tochondrial oxidant stress depletes GSH and can also induce li-pid peroxidation [172]. Additionally, mitochondrial β-oxidation can be inhibited by VPA [156]. This results in carnitine depletion in human skin fibroblast [173]. Thus, VPA decreases level of carnitine in the blood [174].

While exact mechanisms involved in VPA induced carni-tine depletion is not clear [175], several mechanisms have been proposed including formation of valproylcarnitine, reduction of tubular reabsorption of acylcarnitine and carnitine biosynthesis [175]. Besides, decrease of carnitine, VPA treatment can also cause high ammonia plasma level. The urea cycle is the major pathway for ammonia metabolism in the liver. VPA inhibits the mitochondrial enzyme carbamoyl phosphate synthetase I, which is the first enzyme in the urea cycle pathway [176,177]. This impairs the ability of the liver to metabolize and excrete ammonia. The results are high plasma concentration of ammonia. Furthermore, a strong correlation has been established between deficiency of carnitine and reduced mitochondrial bioenergetic function [176]. VPA was shown to deplete carnitine and inhibits the urea cycle. This increases susceptibility to mitochondrial damage. Pharmacological replenishment of the liver carnitine pool is beneficial [178], and it has been suggested to protect against VPA-induced mitochondrial toxicity [179].

### 5.5. Carbamazepine andphenytoin

Carbamazepine and phenytoin can both inhibit mitochon-drial respiration [159,170] and a study with carbamazepine in epileptic children who started new AED drug treatment showed significant reduction of ATP production [149] and mitochondrial respiration [159]. While investigating carbamazepine, pheny-toin and phenobarbital effects on mitochondrial function in a murine hepatic microsomal system used to produce AED metabolites, it was found that mitochondrial membrane potential was also reduced [159]. In the same study, the drugs also impaired Ca^2^ + uptake/release [159]. An evaluation of the cytotoxic me-chanisms of phenytoin in rat hepatocytes showed elevation in ROS formation, depletion of intracellular reduced glutathione, increase in cellular oxidized glutathione, enhancement of lipid peroxidation, and mitochondrial damage [180].

### 5.6. Risk factors of AED-mediated mitochondrial dysfunction

Many risk factors predispose epileptic patients to idiosyn-cratic drug-induced mitochondrial dysfunction [155,181,182]. These risk factors are typically drug-specific and determination of clinical manifestations for diagnosis and prognosis of hepa-totoxicity in a particular patient is difficult [183]. Yet, it is well documented that complex interactions between genetic and metabolic risk factors contribute to mitochondrial dysfunction in drug-induced liver injury [182]. The paramount concern is that mitochondrial disease and identification of mutations in the DNA polymerase-gamma gene are associated with mtDNA depletion/deletion and mitochondrial oxidative phosphorylation disease [149,156]. Thus, underlying mitochondrial diseases are a major risk factor that predisposes epileptic patients under AED treatment to hepatotoxicity. Until a clear mechanism of liver toxicity is discovered, clinical treatment for epilepsy with car-bamazepine, phenytoin, valproic acid and barbiturate, must be completely avoided for patients at risk of developing hepatotoxicity [156]. Safer drugs with a similar pharmaceutical benefit at managing epilepsy should be used [156].

## 6. Amiodarone hepatotoxicity

Amiodarone (AMD) is an anti-arrhythmia medication used to treatand preventvarious types ofirregularheartbeat. Though amiodarone is one of the most frequently prescribed anti-arrhythmics in the United States, it has significant side effects including hepatotoxicity and liver cirrhosis [184]. AMD consists of a benzofuran ring (ring A) coupled to a p-OH-benzene structure substituted with 2 iodines and a diethyl-ethanolamine side chain (ring B) [185], and analysis of the chemical moieties within amiodarone responsible for mitochondrial toxicity indicates that the benzofuran structure is responsible for mitochondrial toxicity of AMD and the presence of iodine is not essential for this function. [185]. Amiodarone was found to have significant detrimental effects on mitochondrial function and liver injury in an in vivo mouse model. This toxicity was found to correlate with levels of the AMD metabolite desethylamiodarone, with increase in plasma triglycerides as well as a decrease in the ratio of reduced/oxidized glutathione in the liver. These changes were suppressed by inhibition of Kupffer cell function, suggesting a role for Kupffer cell activation in AMD-induced liver injury [186]. In a rat model of hepatotoxicity, AMD was shown to increase liver mitochondrial hydrogen peroxide formation and induce cardiolipin peroxidation, accompanied by inhibition of mitochondrial complex I activity and uncoupling of oxidative phosphorylation with decrease in liver ATP levels. These chan-ges were prevented by NAC, implicating free radicals as central mediators of AMD-induced liver injury [187]. Amidarone induced a concentration-dependent increase in cell death in HepG2 cells and isolated rat hepatocytes, accompanied by a significant decrease in respiratory control ratio and oxidation of palmitate in isolated rat liver mitochondria [188]. ROS in hepatocytes were also elevated reiterating their role in induction of cell death [188]. Studies using isolated hamster liver mitochondria showed that a concentration dependent decrease in mitochondrial membrane potential on treatment with AMD, without evidence of thiobarbituric acid-reactive substances formation, suggesting that AMD brings about its effect independent of lipid peroxida-tion in vitro [189]. Interestingly, however, while in vitro studies by the Pessayre group showed that exposure of isolated rat liver mitochondria to AMD resulted in increased mitochondrial ROS formation, use of ethane exhalation as an index of lipid peroxida-tion in vivo showed elevations by 24 hours after administration of AMD to rats [190]. Studies by the Pessayre laboratory have also found that AMD inhibited mitochondrial beta-oxidation of fatty acids and produced microvesicular steatosis of the liver in mice [191]. Using isolated mouse liver mitochondria, they demonstrated a dual effect of AMD on mitochondrial respiration;an initial protonophoric uncoupling effect, followed by inhibi-tion of the respiratory chain at the levels of complex I and complex II [192].

## 7. Additional factors influencing effects of drugs on mitochondrial function

Drug-drug interactions, alcohol and genetic susceptibility (as alluded to earlier for AEDs) are known to affect hepatotoxicity of a number of the drugs discussed above, but the majority of these interactions have been studied in the context of pharmaco-kinetics and drug metabolism. While scarce, a few studies have examined the role of components which could influence hepatotoxicity of some of these drugs. Caffeine has been shown to influence APAP-induced liver injury by improving mitochondrial function [193], thoughit was given 30 minutes priorto APAP treatment, and hence it is unclear if influence on APAP metabolism played a role in the observed protection. Early studies on acute ethanol administration before APAP overdose showed a preventive effect of ethanol on APAP-induced hepatotoxicity due to interference with APAP metabolism [194], though additional mechanisms influencing mitochondrial transport ofNADPH are also suggested [195]. However, studies in chronic ethanol consumption using the Lieber-DeCarli diet in rats showed that high-dose ethanol potentiated APAP hepatotoxicity in liver slices through induction of CYP2E1 in the short term, while long term treatment potentiated it by CYP2E1 induction and selective mitochondrial GSH depletion. [196,197]. This indicates varia-ble effects of ethanol on APAP-hepatotoxicity depending on duration of ethanol consumption as well as dose. Studies on genetic susceptibility to drug induced liver injury have also focused on polymorphisms influencing predominantly drug metabolism or transport, as in the case of the study examining NAT2 acetyla-tor status in prediction of isoniazid-induced liver injury patients [198] or genetic polymorphisms of SLCO1B1, CYP2E1 and UGT1A1 inanti-TB drug-induced hepatotoxicity [199]. While scarce, studies focused on mitochondrial gene polymorphisms have shown that valproic acid modulates mitochondrial and cy-tosolic calcium levels differently in cells expressing two mito-chondrial DNA (mtDNA) polymorphism sites, 8701 and 10398, that alter intracellular calcium signaling and mitochondrial pH [200]. Also, studies examining the association between risk of developing drug-induced liver injury and the common genetic variants of the manganese superoxide dismutase (SOD2 Val16Ala) and glutathione peroxidase (GPX1 Pro200Leu) genes showed that patients homozygous for the SOD2 Ala allele and the GPX1 Leu allele are at higher risk of developing cholestatic DILI [201]. SOD2 Ala homozygotes were also found to be more prone to suffer DILI from drugs that target mitochondria or produce reactive intermediates [201]. This re-iterates the importance of SOD2, illustrated by data showing exacerbation of APAP-induced injury in mice with partial SOD2 deficiency [22,23] and protection offered by the SOD-mimetic mito-tempo in vivo [24].

## 8. Herbal hepatotoxicity

Herbs and other natural products contain a broad spectrum of chemicals with beneficial properties when used in appropriate amounts but toxic features when consumed in excess. Herbal products as medications have been used even before recorded history in Ancient China, India and Egypt. In recent years, the worldwide use of medicinal herbs expanded significantly [202204], and in the United States, the total estimated retail sales of herbs and natural products increased from $4,230 million in 2000 to $6,032 million in 2013, corresponding to a 3.3% annual growth according to the data of the American Botanical Council [205]. This increased use is supported by a general belief among the public that due to the natural source of these products, they are harmless; this belief is, however, inaccurate and mistaken, and there is currently growing evidence showing that herbs and other natural products may cause liver injury, occasionally severe enough to necessitate liver transplantation [206]. Herbal medicine-related hepatotoxicity represents the second most common cause of drug-induced liver injury in Western countries. In the United States, the Drug-Induced Liver Injury Network (DILIN), which studies liver toxicity related to the use of conventional medications or herbal supplements, constitutes the largest database of herbal medicine-related hepatotoxicity. Between 2004 and 2013, among 839 patients who had suffered DILI, herbal dietary supplement was identified as the culprit in 130 cases (15.5%) [207,208].

A large number of herbal medicines have the potential to cause liver injury. For example, the herbal supplements used as performance enhancing agents, which contain steroids, show significant hepatotoxicity [209]. Hydroxycut, a multi-ingredient product used as a stimulator of basic metabolism and a lipoly-tic agent, was eventually recalled from the market by the manufacturer in 2009, as severe hepatocellular injury, leading to liver transplantation and even death, had been associated with this product [210]. Hydroxycut was subsequently reformulated and returned to market, with all new ingredients except caffeine, illustrating the challenge in establishing a causal relationship between the use of herbal medicines and liver injury. Even when highly suspected, direct correlation between a single causative agent and hepatotoxicity can be extremely difficult to prove. Several reasons may account for this. First, herbal supplements may contain several different compounds (some ingredients often unlabeled), and it is hard to identify the specific compound that caused the liver damage. Second, according to weather and harvest conditions, the composition of herbal products may be variable, making identification of the hepatotoxic agent even harder. Third, contamination or adulteration may impede diagnostic approaches and establishment of causality [211].

As it is difficult to identify specific compounds in herbal preparations that cause liver damage and some of the damage is idiosyncratic, relatively few studies focus on mechanisms of liver damage caused by herbal supplements, and fewer still ex-amine the effect of specific ingredients on organelles such as mitochondria to decipher the cause of liver injury. However, mitochondria-mediated apoptosis is a major pathway implica-ted in liver injury caused by herbal supplements in general, and information is available on the mitotoxicity of specific consti-tuents such as pyrrolizidine alkaloids and neo-clerodane diter-penes. Hence, we take these two types of herbal constituents as examples to dissect their role in the mitochondria-mediated apoptotic pathway.

### 8.1. Pyrrolizidine alkaloids

Pyrrolizidine alkaloids (PAs), which are found in a wide variety of plant species of different families worldwide, can cause hepatocyte apoptosis [212]. Currently, more than 660 types of isolated PAs and their nitrogen oxide derivatives have been identified in over 6000 plants [213], and more than half of PAs produce toxicity [214]. Though great progress has been made during the past decade, mechanisms of PA toxicity are not fully understood. It is generally considered that PAs exhibit hepato-toxicity via their activated metabolite, pyrrolic ester, formed by a reaction catalyzed by the hepatic cytochrome P450 (CYP450) enzyme system. This metabolite may reduce GSH levels causing incre-ased oxidant stress and forming adducts with DNA and proteins [215,216]. PAs can also decrease the anti-apoptotic protein Bcl-xL and increase the pro-apoptotic protein Bax. There are two main types of toxic PAs, retronecine and otonecine type [217], which may share similar hepatotoxic signals. For example, a serious of studies conducted by Wang and coworkers showed that both clivorine (an otonecine-type PA) and senecionine (a retronecine-type PA) can induce apoptosis in hepatocytes [218-220]. Clivorine is abundant in *Ligularia hodgsonii* Hook and *Li-gularia dentat* Hara herbs which have been used traditionally for treatment of cough, hepatitis and inflammation in Chinese medicine [212,221]. Senecionine is abundant in plants of the genus *Senecio* (Compositae) in Asia, Europe, North America and other regions [218,222-224]. Clivorine and senecionine both caused hepatotoxicity by shared signaling pathways, in general causing mitochondrial cytochrome c release, activation of caspase-3 and -9, and decreasing anti-apoptotic Bcl-xL to cause hepatocyte apoptosis. Mitochondrial oxidant stress caused by the PAs, resulting in MPT pore opening may account for the mitochondrial protein release. This was confirmed by use of pan-caspase inhibitors and inhibitors of caspase-3 and caspase-9 as well as NAC (an anti-oxidant), which significantly inhibited apoptosis induced by the two PAs [218-220].

### 8.2. Neo-clerodane diterpenes

Wild germander belongs to the Labiatae family and has been used as a folk medicine for several conditions, ranging from the treatment of rheumatism to use as a diuretic and antiseptic agent [225]. However, wild germander can also cause hepato-cyte apoptosis. The use of preparations containing germander were prohibited as capsules containing either germander powder alone, or in combination with camellia tea, which were marketed for use in weight control, caused 26 cases of hepatotoxicity in France [226]. Neo-clerodane diterpenes present in germander power, which can be transformed by hepatic CYP3A to reactive electrophiles are suggested to be involved in the initiation of hepatocellular injury caused by germander, and these reactive metabolites can deplete thiols and increase cellular Ca^2^ + levels [227-229]. This increased calcium may open the MPT pore, cause mitochondrial protein release, caspase activation, and he-patocyte apoptosis. In addition to germander, the herbal medicine skullcap, which is used in anxiety disorders, causes hepato-cyte apoptosis [230-232]. Skullcap also contains neo-clerodane diterpenes, which are transformed by CYP3A into reactive metabolites, which deplete GSH and cellular thiols, increase cytosolic Ca^2^ +, induce the MPT pore opening, resulting in mitochondrial cytochrome c release, caspase activation, and hepatocyte apop-tosis [232].

### 8.3. Effect of herbal constituents on mitochondrial permeability transition and influence on mitophagy

In general, MPT pore opening is the key event in hepatocyte apoptosis caused by herbs with three mechanisms contributing: First, the herbal reactive metabolite may cause DNA damage, which may increase levels of p53 and Bax, which interacts with the MPT pore protein structure and cause MPT pore opening [233]. Second, the herbal reactive metabolite may deplete GSH and cause the oxidation of protein thiols, which may form disul-fide bonds in the MPT pore protein structure and open the MPT pore [234]. Third, the increased cytosolic Ca^2^ + caused by herbal reactive metabolites can increase intra-mitochondrial Ca^2^ +, which is a powerful stimulus for pore opening [235].

Mitochondrial biogenesis is the process of formation of new mitochondria in either physiological or pathophysiological contexts to replenish hepatocyte organelles and maintain cellular ho-meostasis. Biogenesis functions along with processes such as mitochondrial fusion, fission and mitophagy (targeted removal of senescent mitochondria by autophagy) to maintain the cells' metabolic functions. In addition to compromising mitochondrial function as described above, herbs can also affect mitophagy and mitochondria biogenesis to cause hepatotoxicity. The traditional Chinese medicine Herb Ephedra sinica has been shown to induce mitophagy and inhibiting mitochondria biogenesis [236]. The principal active constituents in Ephedra sinica (also known as Chinese ephedra or Ma Huang) are alkaloids which are identical to ephedrine and pseudoephedrine [237] and ephe-drine is widely used as the main substance in weight-loss formulations [238]. The sale of Ephedra containing supplements was banned in 2004 due to cardiotoxicity [239]. Currently accumulating clinical cases also show that ephedrine has potential hepatotoxicity [240,241], with mitochondria playing a key role in ephedrine-induced liver damage. Ephedrine can incre-ase intracellular ROS production in hepatic stellate cells, which may induce the loss of mitochondrial membrane potential, causing accumulation of PINK1 on outer membranes of damaged mitochondria and activating mitophagy [236]. In addition, ep-hedrine can also inhibit mitochondria biogenesis by inducing a decrease in mtDNA [236].

## 9. Mitochondrial biomarkers in drug-induced liver injury

An important aspect of studying the role of mitochondria in drug-induced liver injury is the question if these mechanisms also apply to the human pathophysiology. Acute liver injury and liver failure results in impairment of blood coagulation, which is a contraindication for liver biopsies. Therefore, during the acute phase of liver injury only plasma biomarkers are available that may inform on cell injury mechanisms.

During the last 10 years, a large number of different biomar-kers have been identified and their relevance investigated. Some biomarkers such APAP-protein adducts can assist in diagnosing the likely cause of the liver injury and the severity of the overdose even several days after the poisoning [242]. This led to the recent development of a point-of-care test for the accelerated diagnosis of APAP overdose in patients [243]. Other bio-markers such as miR-122 [244-247], full-length cytokeratin-18 [247-250], high mobility group box 1 (HMGB1) protein [247-249,251], and argininosuccinate synthetase (ASS) [252] are generally considered indicators of mainly necrotic cell de-ath [253]. On the other hand, increased plasma levels of the caspase-cleaved form of cytokeratin-18 [247,248,250, 254, 255] and activated caspases detected as enzyme activity and/or the protein by western blotting [164,247,249,254], are markers of apoptotic cell death. However, due to the sensitivity of the assay, cleaved cytokeratin-18 levels should always be compared to the full-length version in order to assess the relative importance of apoptosis [247,248,254]. The clinical utility of some of these biomarkers may be that they are highly specific for he-patocyte cell death, e.g. miR-122, and that they are even more sensitive indicators of cell injury than the routine parameters of liver cell death plasma ALT and AST activities [252,256]. The enhanced sensitivity is not only reflected in an earlier increase in the plasma levels of these biomarkers but also in a more rapid decline [248,252], which makes most of these compounds more accurate indicators of the time course of acute cell death compared to ALT and AST [253]. Another use of biomarkers such as plasma miRNA profiles can be used to distinguish between clinical conditions that are very similar such as acute liver injury after an APAP overdose and hypoxic hepatitis [257]. However, the clinical application of these parameters is currently limited as the time to obtain the information is generally too long to impact patient treatment.

In addition to the general biomarkers for cell death, some bi-omarkers have been used to investigate the mechanisms of cell injury in humans. As discussed, APAP hepatotoxicity involves mitochondrial dysfunction [163]. In order to evaluate if there is evidence for mitochondrial damage in humans after APAP overdose, plasma of these patients was analyzed for mtDNA and glu-tamate dehydrogenase (GLDH), which are both located in the mitochondrial matrix [164]. In addition, mitochondrial inter-membrane proteins such as endonuclease G are released from damaged mitochondria after APAP and translocate to the nucleus where they cause DNA fragmentation [69]. Thus, nuclear DNA fragments in plasma can be considered indirect biomar-kers of mitochondrial dysfunction at least for APAP overdose [164]. These mitochondrial damage biomarkers could be readily detected in plasma of patients with APAP overdose and hypoxic hepatitis and in APAP-treated mice in a time course that correlated with ALT increases [164,247]. The only substantial difference was the fact that these biomarkers had a shorter half-life in plasma compared to ALT suggesting again that these types of biomarkers reflect more accurately the injury process. However, these biomarkers were not detected in a mouse model of furosemide-induced liver injury, which does not involve mi-tochondrial damage [258] despite severe plasma ALT elevations and hepatocellular necrosis [164]. Thus, these biomarkers reflect more an injury mechanism than just cell death. Interestingly, when comparing a large group of patients who survived an APAP overdose with patients who did either not survive or needed a liver transplant, the surviving patients showed on average less mitochondrial damage as indicated by biomar-kers GLDH, mtDNA and nuclear DNA fragments than the non-surviving patients despite the fact that both groups had similar peak ALT values [259]. These data suggest that mitochondrial injury does not just occur after APAP but is critically involved in the injury process [260]. Another hepatic mitochondrial matrix protein, carbamoyl phosphate synthetase-1 (CPS1), was detected during Fas-induced apoptosis and APAP-induced necrosis in cell culture supernatants, in mice *in vivo* and in plasma of human acute liver failure patients [261]. Similar to mtDNA, GLDH and nuclear DNA fragments [164,247], CPS1 appears to be a general biomarker of cell death involving mitochondria, which means that it is detectable in acute liver failure patients with different etiology [261]. In addition, CPS1 has a half-life of 2 h [261]. This makes CPS1 similar to other cell injury biomarkers, which generally disappear from plasma faster than ALT [164,252]. Another liver- and mitochondria-specific biomarker, ornithine carbamoyltransferase (OCT), is a highly sensitive bi-omarker of cell toxicity [262,263]. Interestingly, despite its mi-tochondrial location OCT appears earlier in plasma during cell injury than the cytosolic enzyme ALT [263]. In addition, the relative activity increases in plasma in response to hepatotoxins were higher compared to ALT, which led to the conclusion that OCT is a superior injury biomarker than ALT [263]. However, the OCT response to hepatotoxic drugs was more variable than the ALT response suggesting that OCT may be a marker of the toxicity mechanism [264]. Thus, the general utility of OCT as diagnostic biomarker needs further evaluation especially in patients with acute liver injury.

Most of the previously discussed biomarkers depend on cell lysis to be detectable in plasma. In contrast, long-chain acylcar-nitines, which have been suggested as markers of liver injury and mitochondrial dysfunctions [265], increased before ALT or AST activities after APAP overdose in mice [265-267]. However, acylcarnitine levels did not increase during furosemide toxicity despite the substantial cell necrosis [266]. This indicates that acylcarnitine levels in plasma may indeed reflect mitochondrial dysfunction, not cell death. Interestingly, acylcarnitine levels did not significantly increase during APAP hepatotoxicity in patients despite the evidence that mitochondrial dysfunction is critical for APAP-induced liver injury in patients [164,259,268]. Howe-ver, it was shown that treatment of mice with NAC, which does not only support GSH synthesis but also mitochondrial energe-tics [18], strongly reduced the increase in acylcarnitine levels in mice [266]. Since all patients suspected of an APAP overdose are treated with NAC as standard of care, this may have been the reason for the lack of elevated acylcarnitine levels in our pa-tients [266]. In support of this conclusion, a study comparing early and late presenting patients with delayed NAC treatment showed higher levels of acylcarnitines in the late presenting pa-tients [269]. These findings indicate that long-chain acylcarniti-nes can be biomarkers of mitochondrial dysfunction even in the absence of cell death; however, NAC treatment may interfere with this biomarker.

In summary, assessment of mitochondrial and other biomar-kers has shown promising results in terms of better understanding the mechanisms of drug hepatotoxicity in patients and encouraging findings regarding the prognosis of acute liver failure. Current limitations include the complicated and time-consuming analysis of most of the biomarkers, which limits the utility in the clinical setting at this point. Additional caveats that need to be considered are the limited organ specificity of some biomarkers and whether a biomarker reflects more a mechanism of cell injury specific for certain drugs or if it reflects general cell dysfunction and injury.

## 10. Overall summary

**Figure 1:**
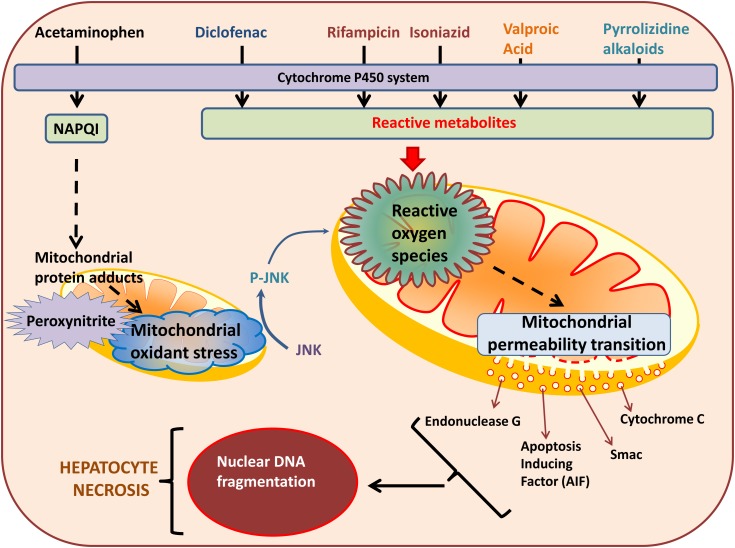


Mitochondria are important targets for drug-induced hepato-toxicity caused by a wide variety of drugs and chemicals. A large number of mitochondrial functions are affected by these various drugs (Table 1), though the common theme in drug-induced mitochondrial dysfunction seems to be elevated production of ROS, which leads to induction of the mitochondrial permeability transition and subsequent cell death (Figure 1). While discrete mechanistic steps during this process have been well studied for drugs such as APAP, this information is lacking for other drugs such as INH and RMP as well as herbal supplements. In addition, recent evidence also indicates that mitochondrial perturbations also influence recovery of hepatic function and this promises to be an area of significant therapeutic importance. From the translational standpoint, non-invasive identification of mito-chondrial specific biomarkers in humans will help confirm the relevance of animal experiments and *in vitro* studies to the clinical context and advances in technology will probably enable more comprehensive analysis of these parameters.

**Table 1:**
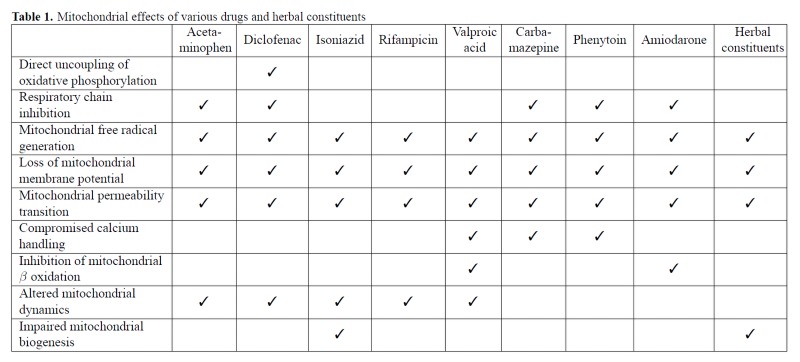


## 11. Future perspectives

In the case of APAP overdose, the importance of mitochondria during the initiation of liver injury is rather well characterized, though intense investigation of discrete steps in the process are ongoing. Unravelling these undoubtedly complex multidimensional steps will provide significant insight into key areas of therapeutic interest. Though the role of mitochondria in regeneration and recovery are now recognized, this area should probably be the main focus for translational research of clinical significance, since late interventions to enhance biogenesis for example will probably have the most therapeutic promise. In the case of anti-epileptic and anti-TB drugs, difficulties in reproducing the clinical situation, where chronic therapeutic treatment is the norm, with animal studies render the information incomplete. For example, with regards to anti-TB drugs, either a short con-centration dependent study is often conducted *in vitro* [119,270] or supra-clinical doses are used *in vivo* [270]. Obviously, these do not replicate the clinical scenario, where only long term chronic therapeutic dosage of anti-epileptic or anti-TB drugs produces hepatotoxicity in humans. Thus, it would be necessary to establish chronic treatment conditions in animal studies and then examine mechanisms of hepatotoxicity and the role of mitochondrial oxidative stress and ROS formation induced by these drugs. For drugs such as diclofenac, though exact mechanisms of hepatotoxicity are unclear, it is evident that the parent compound, as well as its reactive metabolites significantly affects mitochondrial function and cellular ATP production. However, exact mechanisms of how mitochondrial function is affected and whether these are direct effects on the mitochondria or mediated through intermediate cellular signaling is still unclear and needs further study. With regards to herbal supplements, it is now well recognized that hepatotoxicity induced by these compounds involves the mitochondria, though again, insight into mechanistic aspects are scarce. In particular whether mitochondrial alterations are a cause or effect of upstream events needs to be elucidated. In addition to the challenges to study drug hepatotoxicity in more detail in appropriate animal models, a key issue is whether these mechanisms actually translate to the human conditions. A number of mechanistic biomarkers have been established in human APAP hepatotoxicity, the applications of these biomarkers to many other drug-induced liver injury conditions remains to be investigated. Only a comprehensive approach using appropriate *in vitro* models and animal studies together with translational investigations in humans will lead to the discovery of clinically viable therapeutic approaches.
